# Impact of hypertension on arterial stiffness and cardiac autonomic modulation in patients with peripheral artery disease: a cross-sectional study

**DOI:** 10.31744/einstein_journal/2021AO6100

**Published:** 2021-11-24

**Authors:** Breno Quintella Farah, Gabriel Grizzo Cucato, Aluísio Andrade-Lima, Antonio Henrique Germano Soares, Nelson Wolosker, Raphael Mendes Ritti-Dias, Marilia de Almeida Correia

**Affiliations:** 1 Universidade Federal Rural de Pernambuco Recife PE Brazil Universidade Federal Rural de Pernambuco, Recife, PE, Brazil.; 2 Northumbria University Newcastle upon Tyne United Kingdom Northumbria University, Newcastle upon Tyne, United Kingdom.; 3 Universidade Federal de Sergipe Aracajú SE Brazil Universidade Federal de Sergipe, Aracajú, SE, Brazil.; 4 Universidade de Pernambuco Recife PE Brazil Universidade de Pernambuco, Recife, PE, Brazil; 5 Hospital Israelita Albert Einstein São Paulo SP Brazil Hospital Israelita Albert Einstein, São Paulo, SP, Brazil.; 6 Universidade Nove de Julho São Paulo SP Brazil Universidade Nove de Julho, São Paulo, SP, Brazil.

**Keywords:** Intermittent claudication, Peripheral arterial disease, Comorbidity, Cardiovascular system, Vascular stiffness, Hypertension

## Abstract

**Objective:**

To examine the impact of hypertension on cardiovascular health in patients with symptomatic peripheral artery disease and to identify factors associated with uncontrolled hypertension.

**Methods:**

A cross-sectional study including 251 patients with symptomatic peripheral artery disease (63.9% males, mean age 67±10 years). Following hypertension diagnosis, blood pressure was measured to determine control of hypertension. Arterial stiffness (carotid-femoral pulse wave velocity) and cardiac autonomic modulation (sympathovagal balance) were assessed.

**Results:**

Hypertension was associated with higher carotid-femoral pulse wave velocity, regardless of sex, age, ankle-brachial index, body mass index, walking capacity, heart rate, or comorbidities (ß=2.59±0.76m/s, b=0.318, p=0.003). Patients with systolic blood pressure ≥120mmHg had higher carotid-femoral pulse wave velocity values than normotensive individuals, and hypertensive patients with systolic blood pressure of ≤119mmHg (normotensive: 7.6±2.4m/s=≤119mmHg: 8.1±2.2m/s 120-129mmHg:9.8±2.6m/s=≥130mmHg: 9.9±2.9m/s, p<0.005). Sympathovagal balance was not associated with hypertension (p>0.05).

**Conclusion:**

Hypertensive patients with symptomatic peripheral artery disease have increased arterial stiffness. Arterial stiffness is even greater in patients with uncontrolled high blood pressure.

## INTRODUCTION

Peripheral artery disease (PAD) affects more than 200 million people worldwide.^(1)^ Hypertension is one of the most prevalent risk factors for PAD, affecting approximately 80% of patients.^(2–4)^ It is directly related to fatal and nonfatal cardiovascular events in this patient population.^(5)^.

Blood pressure control (*i.e.*, systolic blood pressure <140mmHg and diastolic blood pressure <90mmHg) is thought to be a cornerstone of hypertension management.^(6)^ In fact, a prior study^(7)^ showed that blood pressure control can decrease the incidence of cardiovascular disease and overall mortality by 33% (from 3.85% to 2.59% per year) and 32% (from 2.63% to 1.78% per year), respectively. However, associations between hypertension control and cardiovascular function in patients with PAD remain to be determined.

Deeper understanding of the impact of controlled and uncontrolled hypertension on cardiovascular health may assist physicians in managing cardiovascular health, with potential contributions to the survival of patients with PAD. Factors associated with uncontrolled hypertension in patients with PAD are worthy of investigation.

## OBJECTIVES

To examine the impact of hypertension on cardiovascular health in patients with symptomatic peripheral artery disease, and to identify factors associated with uncontrolled hypertension.

## METHODS

### Recruitment and patients

This cross-sectional study followed (Strengthening the Reporting of Observational studies in Epidemiology (STROBE) checklist.^(8)^Patients with PAD were recruited from vascular units in São Paulo, SP, Brazil. Inclusion criteria were patients aged 40 to 90 years with symptomatic PAD (ankle-brachial index ≤0.90) in one or both legs, absence of critical limb ischemia, rest pain, noncompressible vessels, no limb amputation, or ulcers. Compliance with study criteria was checked by preliminary evaluations.

This study was approved by the Human Research Ethics Committees of *Hospital das Clínicas, Faculdade de Medicina, Universidade de São Paulo*(HCFMUSP), protocol 3.986.124, CAAE: 42379015.3.3002.0068 and of *Hospital Albert Einstein*(HIAE), protocol 3.959.548, CAAE: 42379015.3.0000.0071. Patients were duly informed of study risks and benefits and gave their written informed consent for participation. Data collection was performed between September 2015 and December 2017.

### Cardiovascular measurements

Measurements were made in a quiet environment. Blood pressure and arterial stiffness were measured with patients in the seated and the supine position, respectively. Patients were instructed to avoid moderate to vigorous physical activity for a minimum of 24 hours prior to the visit, and to avoid smoking, alcohol and caffeine intake for a minimum of 12 hours prior to measurements. Data were collected by blinded investigators.

### Hypertension (predictors)

Patients taking antihypertensive drugs and with systolic blood pressure ≥140mmHg or diastolic pressure ≥90mmHg were defined as hypertensive. Blood pressure was measured in both arms using an automatic device HEM-742-E (Omron Corporation, Kyoto, Japan). Three consecutive blood pressure readings were taken at one-minute intervals, as described elsewhere.^(9)^Higher readings were used in the analysis. Patients systolic blood pressure <140mmHg and diastolic blood pressure <90mmHg were allocated to the controlled group. Patients with blood pressure values higher than those established for the controlled group were allocated to the uncontrolled group.^(6)^Intraclass correlation coefficient for systolic and diastolic blood pressure was 0.85 and 0.92, respectively.^(10)^

### Arterial stiffness (outcome)

Carotid–femoral pulse wave velocity (cfPWV) was used to assess arterial stiffness. Measurements were made using a high-fidelity applanation tonometer (Sphygmocor, ATCOR Medical, Australia), in compliance with Clinical Application of Arterial Stiffness Task Force III^(11)^ and American Heart Association Scientific Statement: Recommendations for Improving and Standardizing Vascular Research on Arterial Stiffness^(12)^ guidelines. The intraclass correlation coefficient for cfPWV was 0.91.^(10)^

### Cardiac autonomic modulation (outcome)

Cardiac autonomic modulation assessment was based on heart rate variability analysis, as per previously described procedures.^(9)^ Inter-beat (RR) intervals were obtained using a heart rate monitor (V800, Polar^®^ Electro, Oulu, Finland); a minimum of five minutes of stationary RR interval data were used. Frequency domain variables were calculated using the autoregressive method. Signals operating at frequencies between 0.04 and 0.4Hz were considered physiologically significant. Low (LF) and high (HF) frequency components were represented by oscillations ranging from 0.04 to 0.15Hz and 0.15 to 0.4Hz, respectively. The LF/HF ratio was defined as the cardiac sympathovagal balance. Analyses were performed using Kubios HRV software (Biosignal Analysis and Medical Imaging Group, Joensuu, Finland). Task Force for Heart Rate Variability recommendations were followed.^(13)^

### Covariates

Demographic data (age and sex), ankle-brachial index, comorbidities (diabetes, coronary artery disease, heart failure, chronic kidney disease, cerebrovascular disease and dyslipidemia), walking capacity, heart rate, and body mass index were assessed at the beginning of the study, using previously described procedures.^(9,14)^ Interarm blood pressure differences >10mmHg were considered to be indicative of upper extremity PAD.^(15)^

### Statistical analysis

Data normality and homogeneity were checked using the Kolmogorov Smirnov and the Levene test respectively. For descriptive statistics, continuous variables were summarized as mean and standard deviation, whereas categorical variables were summarized as relative frequency. Linear regression models were used to investigate associations between cardiovascular variables (arterial stiffness and cardiac autonomic modulation) and hypertension. Crude analyses were performed first, then adjusted for classical confounding variables. Residual analysis was performed and homoscedasticity examined using graphical analysis (scatterplots). Multicollinearity analysis was conducted assuming variance inflation factors of less than than 5, and tolerance values of less than 0.20.

One-way ANOVA was used to compare cfPWV values between normotensive and hypertensive patients (≤119mmHg; 120-129mmHg and ≥130mmHg). Multiple logistic regression was used to determine factors associated with uncontrolled hypertension in patients with PAD. Variables with a p value <0.30 in bivariate analysis were included in the model, and only those with a p value <0.10 retained in the final model. Model goodness-of-fit was assessed using the Hosmer-Lemeshow test.

Pearson’s correlation coefficient was used to examine the relation between systolic blood pressure and cfPWV. The level of significance was set at 5% (p<0.05).

## RESULTS

Out of 261 patients enrolled in this study, 10 were excluded due to missing blood pressure data. The final sample comprised 251 patients with PAD whose data were available for analysis. All patients had moderate PAD, and 24.8% had signs suggestive of upper extremity PAD. Mean body mass index was 27.4±6.3kg/m². Hypertension was diagnosed in 89.6% patients, of whom 50.2% had uncontrolled hypertension. Clinical characteristics of patients with controlled or uncontrolled hypertension are shown in table 1. More frequent use of angiotensin-converting enzyme inhibitor (ACE), older age, and higher systolic and diastolic blood pressure values were more prevalent among patients with uncontrolled hypertension relative to patients with controlled hypertension (p<0.05) (Table 1).

**Table 1:** Comparison of general characteristics of patients with peripheral artery disease and controlled or uncontrolled hypertension

Variables		Controlled hypertension	Uncontrolled hypertension	p value
Clinical and demographic variables				
	Male sex (%)	(53.6)	(46.4)	0.130
	Age (years)	65±10	69±8	0.001
	Ankle-brachial index	0.60±0.19	0.57±0.16	0.245
	Body mass index (kg/m²)	28.0±4.9	27.4±4.7	0.342
	Claudication distance (m)	133±85	125±68	0.465
	Six-minute walk test (m)	326±91	318±85	0.531
	Systolic blood pressure (mmHg)	121±12	158±15	<0.001
	Diastolic blood pressure (mmHg)	69±10	78±10	<0.001
Comorbidities				
	Current smoker (%)	(42.9)	(57.1)	0.334
	Diabetes (%)	(48.4)	(51.7)	0.633
	Dyslipidemia (%)	(53.2)	(46.8)	0.056
	Obesity (%)	(52.6)	(47.4)	0.642
	Coronary artery disease (%)	(55.6)	(44.4)	0.241
	Stroke (%)	(56.1)	(43.9)	0.806
Medications				
	Antiplatelet	(51.2)	(48.8)	0.949
	ACE inhibitor (%)	(64.9)	(35.1)	0.018
	Angiotensin-receptor antagonist (%)	(48.4)	(51.6)	0.579
	Calcium channel blocker (%)	(47.5)	(52.5)	0.481
	Diuretic (%)	(45.5)	(54.5)	0.152
	Beta-blocker (%)	(51.6)	(48.4)	0.924
	Statin (%)	(53.2)	(46.8)	0.137
	Hypoglycemic (%)	(47.7)	(52.3)	0.371
	Peripheral vasodilator (%)	(58.5)	(41.5)	0.219
	ACE inhibitor + diuretic + calcium channel blocker (%)	(53.8)	(46.2)	0.904

Values expressed as median±interquartile range and frequency.

ACE: angiotensin conversion enzyme.

Associations between hypertension and cardiovascular variables are shown in table 2. Hypertension was associated with higher cfPWV, regardless of sex, age, ankle-brachial index, body mass index, walking capacity, heart rate or comorbidities (p<0.05). Hypertension was not associated with cardiac autonomic modulation (p>0.05).

**Table 2:** Association between hypertension and cardiovascular variables in patients with symptomatic peripheral artery disease

Independent variable β(SE)		cfPWV(m/s)		LF/HF	
		β(SE)	B	β(SE)	b
Hypertension (no=0, yes=1)	Crude	1.61 (0.66)	0.184*	-0.70 (0.39)	-0.157
	Adjusted	2.37 (0.87)	0.248*	0.13 (0.85)	0.014

* p<0.05. Model for cfPWV: adjusted for sex, age, ankle-brachial index, body mass index, six-minute walk test, heart rate, diabetes, obesity, coronary artery disease, stroke and dyslipidemia. Model for LF/HF: adjusted for sex, age, ankle-brachial index, body mass index, six-minute walk test, diabetes, obesity, coronary artery disease, stroke, and dyslipidemia.

cfPWV: carotid-femoral pulse wave velocity; LF: low frequency; HF: sympathovagal balance; SE: standard error;

b: standardized beta coefficients.

**Figure 1:**
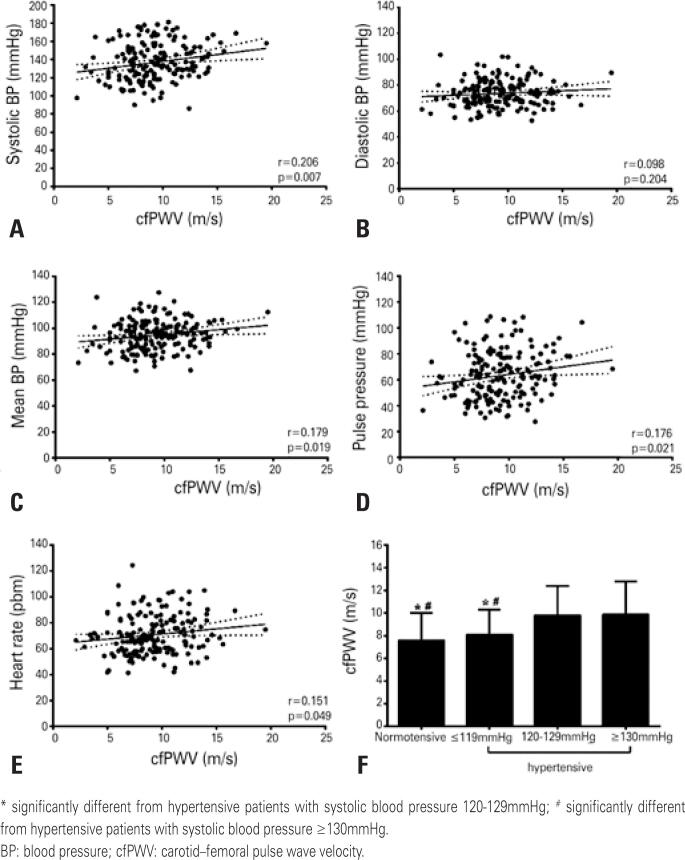
Correlations between systolic blood pressure (A); diastolic blood pressure (B); mean blood pressure (C); pulse pressure (D); heart rate (E) and carotid–femoral pulse wave velocity; comparison of arterial stiffness between normotensive and hypertensive patients with systolic blood pressure of ≤119mmHg, 120-129mmHg, and ≥130mmHg (F)

Systolic blood pressure, diastolic blood pressure, mean blood pressure, heart rate and pulse pressure were positively correlated with cfPWV (Figures 1A-1E). Patients with PAD and systolic blood pressure ≥120mmHg had higher cfPWV than normotensive or hypertensive patients with systolic blood pressure ≤119mmHg (normotensive: 7.6±2.4m/s; systolic blood pressure ≤119mmHg: 8.1±2.2m/s; systolic blood pressure 120-129mmHg: 9.8±2.6m/s; systolic blood pressure ≥130mmHg: 9.9±2.9m/s; p<0.005) (Figure 1F).

Older age, lower ankle-brachial index and no use of ACEi were associated with uncontrolled hypertension, based on regression analysis (Table 3).

**Table 3:** Factors associated with uncontrolled hypertension in patients with peripheral artery disease

Dependent variables	Independent variables	OR (95%CI)	β (SE)	p value
Uncontrolled hypertension (no=0, yes=1)	Age (years)	1.06 (1.02-1.10)	0.061 (0.019)	0.001
	Ankle-brachial index	0.14 (0.02-0.88)	-1.975 (0.941)	0.036
	Use of ACEi (ref=no)	0.38 (0.19-0.76)	-0.970 (0.365)	0.008
	Dyslipidemia (ref=no)	0.42 (0.16-1.05)	-0.878 (0.475)	0.065

Hosmer and Lemeshow test: χ²=7.599; p=0.474.

OR: odds ratio; SE: standard-error; β: regression coefficient; ACEi: angiotensin converting enzyme inhibitor.

## DISCUSSION

In this study, hypertension was associated with greater arterial stiffness in patients with PAD, regardless of sex, age, ankle-brachial index, body mass index, walking capacity, heart rate or comorbidities. Arterial stiffness was also greater in hypertensive patients with PAD and systolic blood pressure ≥120mmHg than in normotensive and hypertensive patients with PAD and systolic blood pressure ≤119mmHg. Older age, lower ankle–brachial index, and no use of ACEi were associated with uncontrolled hypertension in patients with PAD.

Prior studies have shown that hypertension is the most prevalent comorbidity in patients with PAD.^(2–4)^ This analysis revealed similar findings. The prevalence of hypertension was associated with increased arterial stiffness, even after adjustment for confounding factors, as reported in other studies investigating patients with hypertension and diabetes.^(16,17)^ From a clinical standpoint, these are relevant findings, since increased arterial stiffness is associated with poorer cardiovascular outcomes, regardless of traditional risk factors.

Arterial stiffness plays a key role in the pathophysiology of hypertension.^(18,19)^ It is also strongly related to atherosclerosis development and should, therefore, be considered a significant clinical marker in patients with PAD.^(20,21)^ The median cfPWV value was 2.4m/s higher in hypertensive relative to normotensive patients with PAD. This implies a higher cardiovascular risk, given a 1m/s increase in cfPWV is associated with a 14% increase in the odds of having a cardiovascular event and a 15% increase in cardiovascular mortality.^(22)^

Patients with controlled hypertension had lower cfPWV values than those with uncontrolled hypertension, suggesting interventions aimed to reducing cfPWV may benefit patients with PAD. For example, medications with proven ability to decrease arterial stiffening, particularly ACEi and calcium channel blockers,^(23)^ have a positive effect on aortic stiffness. In addition to drug treatment, lifestyle modifications, such as physical activity, should be recommended to reduce blood pressure.

Sympathovagal balance of 2.0 in patients with PAD in this study indicated a shift in cardiac autonomic modulation towards increased sympathetic and decreased parasympathetic modulation. Similar findings have been reported elsewhere.^(24,25)^Increased sympathetic and decreased cardiac parasympathetic modulation are thought to be important predictors of fatal and nonfatal cardiac events.^(26)^ Data in this study failed to reveal a relation between hypertension and sympathovagal balance. This may seem odd at first, since patients with hypertension have an autonomic dysfunction.^(13)^ However, half of patients were taking beta-blockers. Beta-blockers upregulate the fractal behavior of cardiac autonomic modulation in patients with cardiovascular diseases,^(27,28)^ allowing for better cardiovascular control.

In the current study, advanced age, lower ankle-brachial index and no use of ACEi, were associated with uncontrolled hypertension in patients with PAD. Likewise, previous studies revealed lower ankle-brachial index, greater impairment of walking capacity,^(29)^ poor fitness,^(29)^low levels of physical activity,^(30,31)^and more barriers to physical activity, in older patients.^(3,32)^ These variables are classic predictors of good cardiovascular health.^(33)^ Uncontrolled hypertension was also more likely among patients who did not use ACEi. According to a review study,^(34)^ renin-angiotensin system blockers, especially angiotensin converting enzyme inhibitor inhibitors, may effectively reduce the risk of cardiovascular ischemic events in patients with PAD.

Findings of this study have important practical implications. For example, systolic blood pressure <140mmHg and diastolic blood pressure <90mmHg (*i.e.,* controlled blood pressure) are thought to be cornerstones of hypertensive patient management.^(6)^ Hypertension was associated with increased arterial stiffness, regardless of sex, age, ankle-brachial index, body mass index, walking capacity, heart rate or comorbidities. However, hypertensive patients with systolic blood pressure ≤119mmHg had better vascular health (lower arterial stiffness). Therefore, these values should be accounted for in the establishment of therapeutic goals, as suggested in American guidelines,^(35)^is spite of divergences from Brazilian^(6)^ and European guidelines.^(36)^

This study has limitations. Firstly, cross-sectional study design precludes causal inference. Therefore, longitudinal studies are warranted to investigate mechanisms responsible for the associations detected. Secondly, only patients with symptomatic PAD were included. Hence, findings cannot be extrapolated to patients with other stages of the disease. Thirdly, small sample size and the fact that patients were using different drugs may have impacted cardiovascular variables, and stratified analysis according to type of medication was not possible. Finally, biomarkers were not measured, which limits the understanding of mechanisms underlying the associations reported, as well as the extrapolation of findings to other patients.

## CONCLUSION

Hypertension was associated with increased arterial stiffness in patients with PAD. Patients with uncontrolled hypertension had greater arterial stiffness. These findings underscore the significance of blood pressure control in these patients.
