# Supplementary material for: Impact of hypertension on arterial stiffness and cardiac autonomic modulation in patients with peripheral artery disease: a cross-sectional study
Source: Einstein (Sao Paulo). 2021 Nov 24;19:eA06100. doi: 10.31744/einstein_journal/2021AO6100 (PMC8664287; doi:10.31744/einstein_journal/2021AO6100)
Supplement: Translation

# Translation: Impacto da hipertensão na rigidez arterial e modulação autonômica cardíaca em pacientes com doença arterial periférica: um estudo transversal

**Authors:** Breno Quintella Farah, Gabriel Grizzo Cucato, Aluísio Andrade-Lima, Antonio Henrique Germano Soares, Nelson Wolosker, Raphael Mendes Ritti-Dias, Marilia de Almeida Correia

**Affiliations:** 1 Universidade Federal Rural de Pernambuco Recife PE Brasil Universidade Federal Rural de Pernambuco, Recife, PE, Brasil.; 2 Northumbria University United Kingdom Northumbria University, Newcastle upon Tyne, United Kingdom.; 3 Universidade Federal de Sergipe Aracajú SE Brasil Universidade Federal de Sergipe, Aracajú, SE, Brasil.; 4 Universidade de Pernambuco Recife PE Brasil Universidade de Pernambuco, Recife, PE, Brasil; 5 Hospital Israelita Albert Einstein São Paulo SP Brasil Hospital Israelita Albert Einstein, São Paulo, SP, Brasil.; 6 Universidade Nove de Julho São Paulo SP Brasil Universidade Nove de Julho, São Paulo, SP, Brasil.

**Keywords:** Claudicação intermitente, Doença arterial periférica, Comorbidade, Sistema cardiovascular, Rigidez vascular, Hipertensão

## Abstract

**Objetivo:**

Analisar a influência da hipertensão na saúde cardiovascular em pacientes com doença arterial periférica sintomática, e identificar fatores associados à hipertensão arterial não controlada.

**Métodos:**

Neste estudo transversal foram incluídos 251 pacientes com doença arterial periférica (63,9% homens e média de idade 67±10 anos). Hipertensão foi diagnosticada e pressão arterial foi avaliada para determinar o controle da hipertensão. Foram avaliadas rigidez arterial (velocidade da onda de pulso carótida-femoral) e modulação autonômica cardíaca (balanço simpatovagal).

**Resultados:**

Hipertensão foi associada com maior velocidade da onda de pulso carótida-femoral, independentemente do sexo, idade, índice tornozelo-braço, índice de massa corpórea, capacidade de deambulação, frequência cardíaca, ou comorbidades (ß=2,59±0,76m/s, b=0,318, p=0,003). Pacientes com pressão arterial sistólica ≥120mmHg tiveram maior velocidadeda onda de pulso carótida-femoral do que normotensos, e pacientes hipertensos com pressão arterial sistólica ≤119mmHg (normotensos: 7,6±2,4m/s=≤119mmHg: 8,1±2,2m/s 120-129mmHg: 9,8±2,6m/s=≥130mmHg: 9,9±2,9m/s, p<0,005). Balanço simpatovagal não foi associado à hipertensão.

**Conclusão:**

Pacientes hipertensos com doença arterial periférica sintomática apresentam maior rigidez arterial. Em pacientes com pressão arterial não controlada, a rigidez arterial é ainda mais elevada.

## INTRODUÇÃO

A doença arterial periférica (DAP) afeta mais de 200 milhões de indivíduos em todo o mundo.^(1)^ A hipertensão é um dos fatores de risco mais prevalentes para a DAP, e acomete aproximadamente 80% dos pacientes.^(2–4)^ Está diretamente relacionada a eventos cardiovasculares fatais e não fatais nestes pacientes.^(5)^

O controle da pressão arterial (ou seja, pressão arterial sistólica <140mmHg e pressão arterial diastólica <90mmHg) é considerado essencial no tratamento da hipertensão.^(6)^ De fato, estudo anterior^(7)^ mostrou que o controle da pressão arterial pode reduzir a incidência de doenças cardiovasculares em 33% (de 3,85% para 2,59% por ano), e a mortalidade total em 32% (de 2,63% para 1,78% por ano). Entretanto, não se sabe se o controle da hipertensão está associado à função cardiovascular em pacientes com DAP.

Maior compreensão do impacto da hipertensão controlada e não controlada sobre a saúde cardiovascular pode ajudar os médicos em condutas na saúde cardiovascular, o que consequentemente afeta a sobrevida em pacientes com DAP. É importante identificar fatores associados à hipertensão arterial não controlada em pacientes com DAP.

## OBJETIVO

Analisar a influência da hipertensão na saúde cardiovascular em pacientes com doença arterial periférica sintomática, e identificar os fatores associados com a hipertensão não controlada.

## MÉTODOS

### Recrutamento e pacientes

Este estudo transversal segue a lista de verificação *Strengthening the Reporting of Observational studies in Epidemiology* (STROBE).^(8)^ Pacientes com DAP foram recrutados em unidades vasculares em São Paulo, SP, Brasil. Os critérios de inclusão foram pacientes com 40-90 anos de idade com DAP sintomático (índice tornozelo-braço ≤0,90) em uma ou ambas as pernas, e ausência de isquemia crítica de membros, dor ao repouso, vasos não compressíveis, sem membros amputados, ou úlceras. O cumprimento dos critérios de estudo foi verificado por avaliações preliminares.

Este estudo foi aprovado pelo Comitês de Ética em Pesquisa em Humanos do Hospital das Clínicas, Faculdade de Medicina, Universidade de São Paulo (HCFMUSP), protocolo 3.986.124, CAAE: 42379015.3.3002.0068, e do Hospital Albert Einstein (HIAE), protocolo 3.959.548, CAAE: 42379015.3.0000.0071. Cada paciente foi informado dos riscos e benefícios envolvidos no estudo e assinou o termo de consentimento livre e informado para participação. A coleta de dados foi realizada entre setembro de 2015 e dezembro de 2017.

### Mensurações cardiovasculares

Todas as mensurações foram feitas em um ambiente silencioso. A pressão arterial foi verificada com paciente sentado, e a rigidez arterial, na posição supina. Os pacientes foram instruídos a evitar atividade física moderada a vigorosa por, pelo menos, 24 horas antes da visita, e a evitar o fumo, álcool e ingestão de cafeína por, pelo menos, 12 horas antes das mensurações. Os dados foram coletados por pesquisadores cegos para a análise.

### Hipertensão (preditores)

Os pacientes hipertensos foram definidos como aqueles em uso de antihipertensivos e com pressão arterial sistólica de ≥140mmHg ou pressão diastólica de ≥90mmHg. A pressão arterial foi avaliada usando um dispositivo automático HEM-742-E (Omron Corporation, Quioto, Japão). A pressão arterial foi medida três vezes, em ambos os braços, com um intervalo de um minuto entre cada medida, como descrito anteriormente.^(9)^ Para fins do presente estudo, foi utilizada a pressão de braço mais alta. Os pacientes com pressão arterial sistólica <140mmHg e diastólica <90mmHg foram designados para o grupo controlado, enquanto aqueles com valores de PA acima dos indicados para o grupo controlado foram classificados para o grupo não controlado.^(6)^ O coeficiente de correlação intraclasse foi 0,85 e 0,92 para pressão arterial sistólica e diastólica, respectivamente.^(10)^

### Rigidez arterial (resultado)

A rigidez arterial foi avaliada através da medição da velocidade da onda de pulso carotídeo-femoral (cfPWV - *carotid-femoral pulse wave velocity*), usando um tonômetro de aplanação de alta fidelidade (Sphygmocor, ATCOR Medical, Austrália). Foram seguidas as diretrizes da *Clinical Application of Arterial Stiffness Task Force III^(11)^* e da *American Heart Association Scientific Statement: Recommendations for Improving and Standardizing Vascular Research on Arterial Stiffness.*^(12)^ O coeficiente de correlação intraclasse para cfPWV foi 0,91.^(10)^

### Modulação autonômica cardíaca (resultado)

A avaliação da modulação autonômica cardíaca foi obtida através da análise da variabilidade da frequência cardíaca, seguindo os procedimentos descritos anteriormente.^(9)^ Os intervalos inter-batimentos (RR) foram obtidos usando um monitor de frequência cardíaca (V800, Polar^®^ Electro, Oulu, Finlândia); pelo menos cinco minutos de dados de intervalo RR estacionário foram usados. As variáveis de domínio de frequência foram calculadas usando o método autoregressivo. Sinais operando em frequências entre 0,04 e 0,4Hz foram considerados fisiologicamente significativos. Os componentes de baixa frequência (LF - *low frequency*) foi representado por oscilações entre 0,04 e 0,15Hz, e o componente de alta frequência (HF - *high frequency*), entre 0,15 e 0,4Hz. A relação LF/HF foi definida como o equilíbrio simpatovagal cardíaco. Todas as análises foram realizadas utilizando o programa Kubios HRV (Biosignal Analysis and Medical Imaging Group, Joensuu, Finlândia) e seguiram as recomendações da *Task Force for Heart Rate Variability.*^(13)^

### Covariáveis

Dados demográficos (idade e sexo), índice tornozelo-braço, comorbidades (diabetes, doença arterial coronariana, insuficiência cardíaca, doença renal crônica, doença cerebrovascular e dislipidemia), capacidade de deambulação, frequência cardíaca, e índice de massa corporal foram avaliados no início do estudo, utilizando procedimentos previamente descritos.^(9,14)^ A diferença de pressão sanguínea entre braços >10mmHg foi considerada indicativa de DAP dos membros superiores.^(15)^

### Análise estatística

A normalidade e a homogeneidade foram verificadas utilizando os testes de Kolmogorov-Smirnov e Levene, respectivamente. Para a estatística descritiva, as variáveis contínuas foram apresentadas como média e desvio padrão, enquanto as variáveis categóricas como frequência relativa. Os modelos de regressão linear foram utilizados para analisar a associação entre variáveis cardiovasculares (rigidez arterial e modulação autonômica cardíaca) e hipertensão arterial. As análises brutas foram realizadas primeiro e depois ajustadas para as variáveis clássicas de confusão. Foi realizada análise de resíduos residual, sendo a homoscedasticidade analisada pelo gráfico (*scatterplot*). Foi realizada uma análise de multicolinearidade, assumindo fatores de inflação de variância inferiores a 5, e tolerância abaixo de 0,20.

A comparação de cfPWV entre normotensos e hipertensos (≤119mmHg; 120-129mmHg e ≥130mmHg) foi avaliada com a ANOVA *one-way*. A regressão logística múltipla foi usada para identificar fatores associados à hipertensão arterial não controlada em pacientes com DAP. Para este fim, todas as variáveis com p<0,30 na análise bivariada foram incluídas no modelo, mas os critérios para permanecer no modelo final foram variáveis com p<0,10. O teste Hosmer-Lemeshow foi utilizado para avaliar a adequação do modelo.

O coeficiente de correlação de Pearson foi utilizado para analisar a relação entre a pressão arterial sistólica e o cfPWV. O valor de p<0,05 foi considerado estatisticamente significativo.

## RESULTADOS

Dos 261 pacientes inscritos no presente estudo, 10 foram excluídos por não possuírem medida de pressão arterial. A análise final consistiu de dados de 251 pacientes com DAP. Todos os pacientes tinham DAP moderada, e 24,8% tinham indicação de DAP nos membros superiores. O índice de massa corporal médio foi 27,4±6,3kg/m². Hipertensão foi diagnosticada em 89,6% dos pacientes, dos quais 50,2% tinham hipertensão não controlada. A tabela 1 mostra a comparação das características clínicas entre os pacientes com hipertensão controlada e não controlada. Observou-se maior uso de inibidores enzima conversora de angiotensina (ECA), idade mais avançada, e pressão arterial sistólica e diastólica mais alta entre os com hipertensão não controlada do que entre aqueles com hipertensão controlada (p<0,05) (Tabela 1).

**Tabela 1:** Comparação das características gerais da doença arterial periférica com a hipertensão controlada e não controlada

Variáveis		Hipertensão controlada	Hipertensão não controlada	Valor de p
Variaveis clínicas e demográficas				
	Sexo masculino (%)	(53,6)	(46,4)	0,130
	Idade (anos)	65±10	69±8	0,001
	Índice tornozelo-braço	0,60±0,19	0,57±0,16	0,245
	Indice de massa corpórea (kg/m²)	28,0±4,9	27,4±4,7	0,342
	Distância de claudicação (m)	133±85	125±68	0,465
	Teste de de caminhada de 6 minutos (m)	326±91	318±85	0,531
	Pressão arterial sistólica (mmHg)	121±12	158±15	<0,001
	Pressão arterial diastólica (mmHg)	69±10	78±10	<0,001
Comorbidade				
	Fumante atual (%)	(42,9)	(57,1)	0,334
	Diabetes (%)	(48,4)	(51,7)	0,633
	Dislipidemia (%)	(53,2)	(46,8)	0,056
	Obesidade (%)	(52,6)	(47,4)	0,642
	Doença arterial coronariana (%)	(55,6)	(44,4)	0,241
	AVC (%)	(56,1)	(43,9)	0,806
Medicações				
	Antiplaquetários (%)	(51,2)	(48,8)	0,949
	Inibidor da ECA (%)	(64,9)	(35,1)	0,018
	Antagonista do receptor da angiotensina (%)	(48,4)	(51,6)	0,579
	Bloqueador dos canais de cálcio (%)	(47,5)	(52,5)	0,481
	Diurético (%)	(45,5)	(54,5)	0,152
	Beta bloqueador (%)	(51,6)	(48,4)	0,924
	Estatina (%)	(53,2)	(46,8)	0,137
	Hipoglicêmico (%)	(47,7)	(52,3)	0,371
	Vasodilator periférico (%)	(58,5)	(41,5)	0,219
	Inibidor da ECA + diurético + bloqueador dos canais de cálcio (%)	(53,8)	(46,2)	0,904

Os valores são apresentados em mediana±intervalo interquartil e frequência.

AVC: acidente vascular cerebral; ECA: enzima conversora de angiotensina.

A tabela 2 mostra a associação da hipertensão com as variáveis cardiovasculares. A hipertensão foi associada com o maior cfPWV, independente de sexo, idade, índice tornozelo-braço, índice de massa corpórea, capacidade de deambulação, frequência cardíaca, ou comorbidades (p<0,05). Hipertensão não estava associada à modulação autonômica cardíaca (p>0,05).

**Tabela 2:** Associação entre hipertensão arterial e variáveis cardiovasculares em pacientes com doença arterial periférica sintomática

Variável independente β(EP)		cfPWV(m/s)		LF/HF	
		β(SE)	B	β(SE)	b
Hipertensão (não=0, sim=1)	Bruta	1,61 (0,66)	0,184*	-0,70 (0,39)	-0,157
	Ajustada	2,37 (0,87)	0,248*	0,13 (0,85)	0,014

* p<0.05. Modelo para cfPWV: ajustado para sexo, idade, índice tornozelo-braquial, índice de massa corpórea, teste de caminhada de seis minutos, frequência cardíaca, diabete, obesidade, doença arterial coronária, acidente vascular cerebral e dislipidemia. Modelo para LF/HF: ajustado para sexo, idade, índice tornozelo-braço, índice de massa corpórea, teste de caminhada de seis minutos, diabete, obesidade, doença arterial coronária, acidente vascular cerebral, e dislipidemia.

cfPWV: velocidade de onda de pulso carotídeo-femoral; LF: baixa frequência; HF: alta frequência;

EP: erro padrão; b: padronizado coeficientes beta.

Foi observada uma correlação positiva entre pressão arterial sistólica, pressão arterial diastólica, pressão arterial média, frequência cardíaca e pressão de pulso com cfPWV (Figuras 1A-1E). Os pacientes com DAP e pressão arterial sistólica ≥120mmHg apresentaram cfPWV mais alta que pacientes normotensos ou hipertensos com pressão arterial sistólica ≤119mmHg (normotensos: 7,6±2,4m/s; pressão arterial sistólica ≤119mmHg: 8,1±2,2m/s; pressão arterial sistólica 120-129mmHg: 9,8±2,6m/s; pressão arterial sistólica ≥130mmHg: 9,9±2,9m/s; p<0,005) (Figura 1F).

**Figura 1:** Correlação entre pressão arterial sistólica (A); pressão arterial diastólica (B); pressão arterial média (C); pressão de pulso (D) e frequência cardíaca (E), com velocidade de onda de pulso carotídeo-femoral e comparação da rigidez arterial entre pacientes normotensos, hipertensos com pressão arterial sistólica= ≤119mmHg, 120-129mmHg, e ≥130mmHg (F)

Os fatores associados à hipertensão não controlada foram idade avançada, menor índice tornozelo-braço e não uso de iECA com base na análise de regressão logística (Tabela 3).

**Tabela 3:** Fatores associados à hipertensão arterial não controlada em pacientes com doença arterial periférica

Variáveis dependentes	Variáveis independentes	OR (IC95%)	β (EP)	Valor de p
Hipertensão não controlada (não=0, sim=1)	Idade (anos)	1,06 (1,02-1,10)	0,061 (0,019)	0,001
	Índice tornozelo-braço	0,14 (0,02-0,88)	-1,975 (0,941)	0,036
	Uso de iECA (ref=não)	0,38 (0,19-0,76)	-0,970 (0,365)	0,008
	Dislipidemia (ref=não)	0,42 (0,16-1,05)	-0,878 (0,475)	0,065

Teste Hosmer e Lemeshow: χ ²=7,599; p=0,474.

OR: odds ratio; EP: erro-padrão; β: coeficiente de regressão; iECA: inibidor da enzima conversora de angiotensina.

## DISCUSSÃO

Neste estudo, hipertensão estava associada à maior rigidez arterial entre os pacientes com DAP, independente do sexo, idade, índice tornozelo-braço, índice de massa corpórea, capacidade de deambulação, frequência cardíaca ou comorbidades. Rigidez arterial também foi maior em pacientes hipertensos com DAP e pressão arterial sistólica ≥120mmHg, do que em pacientes normotensos e hipertensos com DAP e pressão arterial sistólica de ≤119mmHg. Idade avançada, menor índice tornozelo-braço, e não uso de iECA foram associados com hipertensão não controlada em pacientes com DAP.

Estudos anteriores mostraram que a hipertensão é a comorbidade mais prevalente entre os pacientes com DAP.^(2–4)^ Esta análise mostrou achados semelhantes. A prevalência da hipertensão foi associada a maior rigidez arterial, mesmo após o ajuste para fatores de confusão; como descrito em outros estudos envolvendo pacientes com hipertensão e diabetes.^(16,17)^ Esses achados são relevantes do ponto de vista clínico, pois o aumento da rigidez arterial está associado a piores resultados cardiovasculares, independente dos fatores de risco tradicionais.

A rigidez arterial tem um papel essencial na fisiopatologia da hipertensão,^(18,19)^ e se relaciona fortemente ao desenvolvimento da aterosclerose e, portanto, deve ser considerada um marcador clínico importante em pacientes com DAP.^(20,21)^ O valor mediano de cfPWV foi 2,4m/s maior em hipertensos do que em normotensos com DAP. Isto se traduz em um risco cardiovascular maior, dado que um aumento de 1m/s no cfPWV está associado a um aumento de 14% na probabilidade de ter um evento cardiovascular, e de 15% na mortalidade cardiovascular.^(22)^

Os pacientes com hipertensão controlada tinham valores menores de cfPWV que aqueles com hipertensão não controlada, sugerindo que intervenções para reduzir a cfPWV poderiam ser benéficas para os pacientes com DAP. Por exemplo, a escolha de medicamentos já conhecidos por diminuir a rigidez arterial, particularmente iECA e bloqueadores dos canais de cálcio,^(23)^têm um efeito positivo sobre a rigidez aórtica. Além do tratamento medicamentoso, devem ser recomendadas modificações no estilo de vida, como exercícios físicos, para reduzir a pressão sanguínea.

Demonstramos que o equilíbrio simpatovagal em pacientes com DAP era 2,0, e indicando modulação autonômica cardíaca em direção a maior modulação simpática e menor modulação parassimpática. Achados semelhantes foram descritos em estudos anteriores.^(24,25)^ O aumento da modulação simpática e a redução da modulação parassimpática do coração foram considerados como importantes preditores de eventos cardíacos fatais e não fatais.^(26)^ Os dados do estudo não mostraram uma relação entre hipertensão e equilíbrio simpático. Esta falta de relação pode parecer estranha no início, já que os pacientes com hipertensão têm disfunção autonômica.^(13)^ Entretanto, metade dos pacientes estava tomando beta bloqueadores. Beta bloqueadores regulam positivamente o comportamento fractal da modulação autonômica cardíaca em pacientes com doenças cardiovasculares,^(27,28)^permitindo um melhor controle cardiovascular.

No estudo atual, idade mais avançada, menor índicetornozelo-braço, e não uso de iECA foram associados à hipertensão não controlada em pacientes com DAP. Da mesma forma, estudos anteriores mostraram menor índice tornozelo-braço, maior dificuldade na capacidade de deambulação,^(29)^ pior forma física,^(29)^ baixos níveis de atividade física,^(30,31)^ e mais barreiras à atividade física, em pacientes mais velhos.^(3,32)^Estas variáveis são preditores clássicos de boa saúde cardiovascular.^(33)^ Além disso, foi observado que pacientes que não usavam iECA tinham uma maior probabilidade de hipertensão não controlada. Um estudo de revisão^(34)^ demonstrou que o uso de bloqueadores do sistema renina-angiotensina, especialmente os iECA, pode ser eficaz na redução do risco de eventos isquêmicos cardiovasculares em pacientes com DAP.

Os resultados do presente estudo têm importantes implicações práticas. Por exemplo, pressão arterial sistólica <140mmHg e a diastólica <90mmHg (por exemplo, pressão arterial controlada) são consideradas fundamentais no tratamento de pacientes hipertensos.^(6)^ A hipertensão, independente de sexo, idade, índice tornozelo-braço, índice de massa corpórea, capacidade de deambulação, frequência cardíaca, ou comorbidades, foram associadas à maior rigidez arterial. Entretanto, observamos que pacientes hipertensos com pressão arterial sistólica ≤119mmHg tinham melhor saúde vascular (por exemplo, menor rigidez arterial). Portanto, estes valores devem ser considerados ao estabelecer os objetivos terapêuticos, como sugerido pelas diretrizes americanas,^(35)^ embora com divergências em relação às diretrizes do Brasil^(6)^e europeias.^(36)^

Este estudo tem limitações. Primeiro, desenho transversal de estudo não deixa inferir sobre causalidade. Portanto, estudos longitudinais são necessários para investigar os mecanismos responsáveis pelas associações observadas. Segundo, incluímos apenas pacientes com DAP sintomática; assim, os resultados não podem ser extrapolados para pacientes com outros estágios da doença. Terceiro, o tamanho da amostra era pequeno, e os pacientes estavam usando diferentes medicamentos; isto pode ter influenciado nas variáveis cardiovasculares, e não foi possível fazer análises estratificadas por medicamentos. Finalmente, os biomarcadores não foram medidos, e isso limita a compreensão dos mecanismos que levam às associações observadas, assim como a extrapolação dos resultados para outros pacientes.

## CONCLUSÃO

A hipertensão estava associada à maior rigidez arterial em pacientes com doença arterial periférica. Pacientes com hipertensão não controlada tinham maior rigidez arterial. Estes resultados demonstram a importância do controle da pressão arterial nesses pacientes.
